# Relationship of place of death with care capacity and accessibility: a multilevel population study of system effects on place of death in Norway

**DOI:** 10.1186/s12913-020-05283-6

**Published:** 2020-05-24

**Authors:** Jorid Kalseth, Thomas Halvorsen

**Affiliations:** Department of Health Research, SINTEF Digital, P.O. Box 4760, Sluppen, NO-7465 Trondheim, Norway

**Keywords:** Place of death, End-of-life care, Hospital, Nursing home, Home death, Health system, Health services, Long-term care, Accessibility, Capacity

## Abstract

**Background:**

While the majority of deaths in high-income countries currently occur within institutional settings such as hospitals and nursing homes, there is considerable variation in the pattern of place of death. The place of death is known to impact many relevant considerations about death and dying, such as the quality of the dying process, family involvement in care, health services design and health policy, as well as public versus private costs of end-of-life care. The objective of this study was to analyse how the availability and capacity of publicly financed home-based and institutional care resources are related to place of death in Norway.

**Methods:**

This study utilized a dataset covering all deaths in Norway in the years 2003–2011, contrasting three places of death, namely hospital, nursing home and home. The analysis was performed using a multilevel multinomial logistic regression model to estimate the probability of each outcome while considering the hierarchical nature of factors affecting the place of death. The analysis utilized variation in health system variables at the local community and hospital district levels. The analysis was based on data from two public sources: the Norwegian Cause of Death Registry and Statistics Norway.

**Results:**

Hospital accessibility, in terms of short travel time and hospital bed capacity, was positively associated with the likelihood of hospital death. Higher capacity of nursing home beds increased the likelihood of nursing home death, and higher capacity of home care increased the likelihood of home death. Contrasting three alternative places of death uncovered a pattern of service interactions, wherein hospital and home care resources together served as an alternative to end-of-life care in nursing homes.

**Conclusions:**

Norway has a low proportion of home deaths compared with other countries. The proportion of home deaths varies between local communities. Increasing the availability of home care services is likely to enable more people to die at home, if that is what they prefer.

## Background

The organization of end-of-life (EoL) care in high-income countries has undergone major shifts. Increasing capacity in institutional care during the twentieth century led to fewer home deaths and a corresponding increase in hospital and nursing home deaths [1–3]. While the proportion of nursing home deaths increased, the trend reversed for hospital deaths in countries such as the US, Canada and England, where the proportion of home deaths started to increase towards the end of the twentieth century [4–7]. Norway showed the same trend in hospital and nursing home deaths, partly due to changes in the EoL care for growing patient groups such as patients with cancer, who were increasingly offered this care in nursing home settings instead of hospitals. Moreover, this development coincided with major demographic and epidemiological trends that also led to the continuing decrease in the rate of home deaths in Norway during the first decade of the twenty-first century [8].

People generally wish to die at home [4, 9], but the majority of deaths in high-income countries still occur within institutional settings [10]. While common demographic and epidemiological trends are important determinants in the demand for EoL care [8], there is still considerable variation in how this care is organized [11]. A core value for palliative care has been to enable people to make choices about their EoL care and place of death (PoD) [12]. Some conditions require that EoL care be administered within a hospital setting, while adequate care can be provided more easily in the patient’s home for other conditions. Nevertheless, to what degree the PoD is influenced by availability of institutional or home-based care is an open question. If, for example, resources are steered towards home-based care, does this have the potential to provide more people with EoL care in their homes and eventually increase the proportion of home deaths? The limited existing literature on the relationship between service accessibility, capacity and PoD is divided. Most studies refer to specific subpopulations of people who have died, such as patients with cancer [13], those with dementia [14] or very old persons [15], or to specific settings, i.e. excluding important PoDs such as nursing homes [11]. In addition, the groups compared may be heterogeneous, i.e. when the analyses involve only binomial contrasts [16].

The objective of this study was to analyse how the availability and capacity of home-based and institutional care resources are related to PoD, utilizing a dataset covering all deaths in a national population, i.e. covering all ages and causes of death. We contrasted three PoDs, namely hospital, nursing home and home, while considering the hierarchical nature of factors affecting the PoD. More specifically, we wished to examine how the availability and capacity of care resources in Norwegian municipalities and hospital districts were associated with the PoD, controlling for individual and contextual factors related to the demand for EoL care. Our analysis covered the entire population, i.e. all deaths in Norway, for the period 2003–2011. This approach enabled us to consider both time variation and cross-sectional variation in service availability and capacity.

## Methods

In the analysis of PoD decisions, we decided to build on Andersen and Newman’s [17] framework for health service utilization, which models individual health care consumption as a function of individual characteristics and the characteristics of the environment where the individual lives. The framework separates individual determinants of health care utilization into factors predisposing individuals to certain services such as age and sex, enabling factors such as marital status and income level, and need factors reflecting frailty and the level of illness. Societal determinants (norms and technology) and the health services system are the two major environmental or context dimensions influencing health service utilization. Resources, that is, the labour and capital devoted to providing services, and their organization, i.e. how the resources are controlled and coordinated, are the central elements in the health services system. The resource component involves both the volume of available resources in the system and their geographical distribution. The organization of services comprises accessibility, related to financial aspects as well as to travel times and waiting times, and structure, which relates to characteristics of the system that determine the patients’ journey through the system such as medical practices and referral systems. Norway has a publicly funded health and long-term care system, organized into two administrative levels. The first level comprises primary health care and long-term care (LTC). This includes general practitioners, home-based services and nursing home care and is the responsibility of the municipalities. The second level, hospital services and other specialist health care, is the responsibility of the state and is organized within hospital districts under regional health authorities. EoL care is organized within the regular service system. Service utilization in the last months, weeks and days of life occurs in hospitals, nursing homes or at home.

In line with Andersen and Newman’s [17] framework for health service utilization, we sought to analyse PoD as a response to individual and system characteristics. Local contextual factors, i.e. population characteristics at the municipal level, represent the societal determinants in our model.

Hence, in modelling the choice between PoD in patients’ homes, nursing homes and hospitals, we built on the assumption that PoD decisions can be expressed as a function of patient characteristics, population characteristics of municipalities, the capacity of home-based care and institutional care within municipalities, the capacity of hospital care within hospital districts and the geographical accessibility to services in terms of travel times measured at the municipal level. In the form of an equation, it can be expressed as:

$$ P\left({PoD}_i\right)=f\left({I}_i,{P}_{\mathrm{m}},{C}_m,{C}_h,{A}_m\right), $$

where the discrete individual-level _[*i*]_ probability of PoD depends on a vector of individual characteristics *I* and municipal-*level*_[*m*]_ vectors of population characteristics *P*, municipal service capacities *C* and accessibility *A*. In addition, at the hospital district level _[*h*]_, there is a vector of hospital capacity characteristics *C*.

The variables at each level, the data sources and number of observations are shown in Table 1.

**Table 1:** Variables, data sources and number of observations

**Level**	**Variable**	**Source**	**N** (2003–2011)
**Individual**	PoD (Hospital, Home or Nursing home)	NCoDR	351,907 (2465 missing higher-level variables)
	**Predisposing**		
	Age (six groups)		
	Gender		
	**Enabling**		Analyses: 100*20% samples
	Marital status (four groups)		
	**Need**		
	CoD (six groups)		
**Municipal**	**Population characteristics (context)**	SSB	423–428 (excluding municipalities with missing data)
	- Death rate (per 1000 population)		
	- Age 67–79, 80–89 and 90+ years (% in total population)		
	- Married (% in population aged 18+)		
	- Female employment (> 30 h/week) (% in female population aged 16–66 years)		
	- Population size (five dummy groups)		
	**Accessibility**	*(Travel time to hospital - own data)*	
	- Travel time to municipal centre		
	- Travel time to local hospital		
	- Travel time to local+ hospital (hospital with additional functions)		
	- Travel time to regional hospital		
	**Capacity**		
	- Nurses in LTC (FTP per 100 population aged 67+ years)		
	- Relative size home care (% of total LTC labour costs)		
	- Nursing home beds (per 100 population aged 80+ years)		
**Hospital district**	**Capacity**	SSB	43
	- Hospital beds (somatic beds, per 1000 population)		

*CoD* cause of death, *FTP* full-time positions, *LTC* long-term care, *NCoDR* Norwegian Cause of Death Registry, *PoD* place of death, *SSB* Statistics Norway

The operationalization of the model was restricted by data availability. The analysis was based on data from two public sources: the Norwegian Cause of Death Registry (NCoDR) and Statistics Norway (SSB). Data on the decedents’ PoD, our dependent variable, were collected from the NCoDR. We only considered PoD at home, in nursing homes and in hospitals. ‘Nursing homes’ included all institutional deaths not included in the hospital category. The NCoDR provided all *individual-leve*l characteristics (*I*_*i*_) in the form of year of death, age at death, gender, marital status and main underlying cause of death (CoD). The remaining data were collected from SSB, except for data on travel time to hospitals, which were available from a previous study [18]. *Contextual* variables included municipal population characteristics (*P*_*m*_), such as the death rate, demographic (age) distribution, marriage rates, female employment rates and population size. Health service system resources included volume measures of s*ervice capacity* distributed at the municipal and hospital district levels. Municipal service capacity (*C*_*m*_) included a measure of the home-based care capacity, in the form of labour costs of home-based care as a percentage of total labour costs within municipal LTC, and a measure of institutional care capacity, in the form of the number of nursing home beds per 100 population aged 80+ years. We also included municipal LTC nursing capacity in the form of a count of full-time nursing positions per 100 population aged 67+ years. Our final capacity measure was the (somatic) hospital bed rate at the hospital district level (*C*_*h*_). Service system organization was captured both by including variables for different services at different levels and by including variables reflecting geographical *accessibility*. Average travelling time to the municipal centre was included as a proxy for distance to municipal services. Hospitals may have different functions, and thus we separated them into local hospitals, hospitals with additional speciality functions and regional hospitals. The latter two types also serve as local hospitals for some municipalities. Hospital districts typically include several hospitals. A regional hospital is found in four of the hospital districts. Accessibility to hospital care included four variables: i) whether a hospital is located in the municipality, ii) average travel time from municipal centre to nearest local hospital, iii) nearest hospital with additional speciality functions and iv) nearest regional hospital.

The collected data were structured into four measurement levels. Hospital districts were at the highest level. The hospital districts encompass a varying number of municipalities, which represent the second highest level. Within these two administrative levels, we have repeated yearly measurements (third level), within which the individual-level data were nested (fourth level). Because a multilevel analysis was especially suited to estimate models of hierarchical data structures such as this and because PoD had three discrete outcomes, we selected a multinomial logistic regression model to estimate the probability of each outcome.

Of the approximately 374,000 deaths in Norway in the years 2003–2011, approximately 22,000 were excluded either due to missing PoD (1.5%) or belonging to the category ‘other places’ (4.4%). Another 2465 were excluded due to missing information on municipal-level characteristics. The number of municipalities included from year to year varies from 423 to 428 due to both missing data and municipal mergers. Each municipality was assigned to a hospital district, and we maintained the number of hospital districts as 43 over the period. The municipality of Oslo (the capital) has historically been divided into several acute hospital districts, but because the organization of the hospital catchment areas in the capital area had undergone multiple changes during the study period, we treated the hospitals in the capital area as one. The data had a four-level nested structure (individual, year, municipality and hospital district) and the dependent variable was multinomial (death at home, in hospital and in nursing home). The analyses were performed with both hospital and nursing home as a reference category to capture all three relevant comparisons. Because of the sheer size of our dataset, estimation capacity restraints made it difficult to perform multilevel multinomial regression analysis on the entire sample. Instead, we drew 100 random 20% bootstrap samples (*N* = 69,887) within year, hospital district and PoD. We report the mean results for the relative risk ratio (RRR). The 95% confidence interval for the mean and standard error of the RRR from the 100 regressions were at the mean value or with ±0.01 for all variables except for the mean RRR of home versus nursing home death for the age group 0–49 years, which were within ±0.03. The analyses were performed using MLwiN version 3.00 and STATA/MP 14.0.

## Results

### PoD

**Fig. 1:**
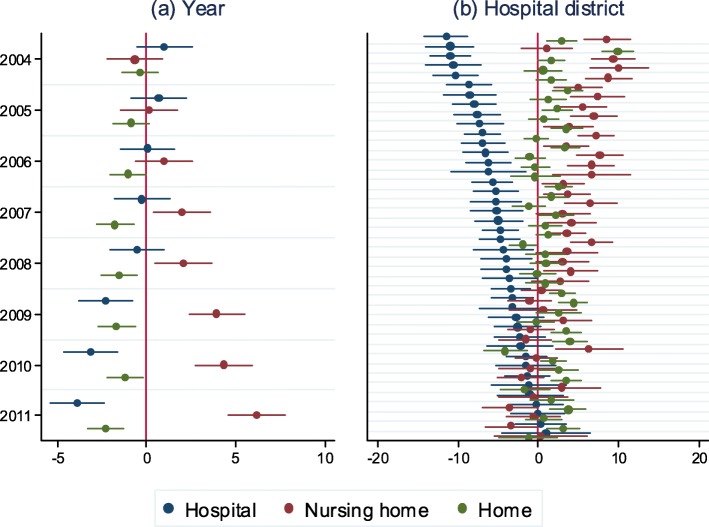
Differences in distribution (%) of place of death in hospital, nursing home and at home at municipal level, by (**a**) year, reference = 2003 (controlling for hospital district), and (**b**) hospital district, reference = capital area (controlling for year)

In the study period, 15.8% of people died at home, 40.1% in hospitals and 44.1% in nursing homes (Table 2). Hence, the majority of deaths occurred within institutional settings. The percentages for different PoDs varied between years, municipalities and hospital districts. Figure 1 shows the differences in the distribution of PoD at the municipal level by year (controlling for hospital district) and by hospital district (controlling for year). The average proportion of nursing home deaths increased, and the proportion of home deaths and hospital deaths decreased over the study period. The average percentage of hospital deaths at the municipal level was more than 10 percentage points higher in the capital area than in the hospital districts with lowest average proportion of hospital deaths.

**Table 2:** Descriptive statistics (decedents’ characteristics, excluding deaths in ‘other places’; years 2003–2011)

	Place of death			
	Home	Hospital	Nursing home	Total
N	55,668	141,091	155,148	351,907
%	15.8	40.1	44.1	100.0
Year % p10–p90	14.9–16.5	36.4–42.5	40.9–48.5	
Municipality % p10–p90	11.9–21.2	27.3–45.7	37.9–56.7	
Hospital district % p10–p90%	13.5–17.9	36.1–42.8	40.6–48.1	
	%	%	%	%
Gender = Women	41.8	47.2	62.0	52.9
Gender = Men	58.2	52.8	38.0	47.1
Age = 0–49 years	10.4	5.7	0.6	4.2
Age = 50–59 years	11.2	7.4	1.6	5.4
Age = 60–69 years	16.8	14.5	4.7	10.5
Age = 70–79 years	22.7	24.9	15.5	20.4
Age = 80–89 years	28.8	37.0	46.9	40.1
Age = 90+ years	10.1	10.6	30.8	19.4
Marital status = Married	36.4	43.6	25.9	34.7
Marital status = Widowed	30.1	34.3	56.3	43.4
Marital status = Divorced	15.0	10.4	7.1	9.7
Marital status = Unmarried	18.5	11.6	10.7	12.3
CoD = Cancer	19.7	33.8	24.4	27.4
CoD = Dementia	2.2	0.6	12.5	6.1
CoD = Circulation	40.3	34.5	34.2	35.3
CoD = Respiratory	7.7	10.1	10.7	10.0
CoD = External	11.8	4.8	2.8	5.0
CoD = Other	18.3	16.2	15.3	16.1

*p10* 10th percentile, *p90* 90th percentile, *CoD* cause of death

### Decedent characteristics

Of the approximately 350,000 decedents included in this study, 53% were women, nearly 60% were aged above 80 years, 43% were widowed, 35% died of diseases of the circulatory system and almost 30% died of cancer (Table 2). The composition of the deceased population differed between the PoDs, with the proportion of old, widowed and women being lowest for home deaths and highest for nursing home deaths.

### Context characteristics

There was substantial variation in the municipal context, in terms of mortality rates, age composition, female work participation and population size. Approximately half of the municipalities had less than 5000 inhabitants, and only 3% had more than 50,000 inhabitants (Table 3).

**Table 3:** Descriptive statistics (municipal and hospital district-level variables; years 2003–2011)

	Mean	Standard deviation
**Municipal level (*N*** **= 3837** [= number of municipalities times number of years]**)**		
Death rate (per 1000 population)	10.5	3.3
Age 67–79 years (per 100 population)	9.8	2.0
Age 80–89 years (per 100 population)	4.7	1.3
Age 90+ years (per 100 population)	0.9	0.4
Married (per 100 population aged 18+ years)	48.6	4.9
Female employment (> 30 h/week) (per 100 women aged 16–66) years)	39.1	6.3
Population 5–99,000	21.0^a^	
Population 10–19,999	13.2^a^	
Population 20–49,999	8.1^a^	
Population 50,000+	3.0^a^	
Travel time to municipal centre (average, minutes)	9.0	6.9
Hospital located in municipality	12.2^a^	
Travel time to local hospital^b^	71.8	68.5
Travel time to local+ hospital^b^	149.5	163.1
Travel time to regional hospital^b^	244.4	181.5
Nurses in LTC (per 100 population aged 67+ years)	3.9	1.4
Relative size homecare (% labour costs)	49.2	13.8
Nursing home beds (per 100 population aged 80+ years)	21.5	8.2
**Hospital level (*N*** **= 387** [= number of hospital districts times number of years]**)**		
Hospital beds (somatic beds, per 1000 population)	2.6	0.8

*LTC* long-term care

^a^Percentage, ^b^Average minutes from municipal centre

### System characteristics

The average travel time to municipal centres was 9 min. The average travel time to the nearest local hospital was 72 min, while the corresponding time to hospitals with specialized functions and to regional hospitals was 2.5 and 4 h, respectively, and 12% of the municipalities hosted a hospital within their borders. There were on average 3.9 nurses per 100 population aged 67+ years in municipal LTC services, 21.5 nursing home beds per 100 population aged 80+ years, and approximately 50% of LTC salary budgets were spent on home care services. Finally, there were on average 2.6 hospital beds per 1000 population in the hospital catchment areas (Table 3).

### Estimation results

Table 4 presents the results from the multinomial regression model, wherein PoD was estimated as the outcome of three contrasts: home/hospital, nursing home/hospital and home/nursing home.

**Table 4:** Multilevel multinomial logistic regression (relative risk ratio^d^)

	Home versus hospital death	Nursing home versus hospital death	Home versus nursing home death
**Individual level**			
*Gender (Ref = Men)*			
Women	0.84^c^	1.32^c^	0.66^c^
*Age (Ref = 90+ years)*			
0–49 years	1.80^c^	0.04^c^	46.30^c^
50–59 years	2.03^c^	0.08^c^	26.4^c^
60–69 years	1.64^c^	0.13^c^	13.43^c^
70–79 years	1.2^c^	0.25^c^	4.73^c^
80–89 years	0.89^c^	0.49^c^	1.82^c^
*Marital status (Ref = Unmarried)*			
Married	0.7^c^	0.57^c^	1.22^c^
Widowed	0.83^c^	0.82^c^	0.99
Divorced	1.02	0.84^c^	1.14^c^
*Cause of death (Ref = Other)*			
Cancer	0.49^c^	1.23^c^	0.37^c^
Dementia	4.08^c^	16.01^c^	0.25^c^
Circulation	1.16^c^	0.86^c^	1.44^c^
Respiratory	0.73^c^	0.99	0.77^c^
External	1.73^c^	0.58^c^	2.54^c^
**Municipal level**			
***Population characteristics (context)***			
Death rate	1.01	1.01	1.00
Age 67–79 years	1.02	1.04^b^	0.99
Age 80–89 years	0.94^a^	0.98	0.97
Age 90+ years	1.08	1.07	1.03
Married	1.00	0.98^c^	1.01^c^
Female employment	0.99^c^	1.01^c^	0.98^c^
Population 5–99,000	0.99	0.99	1.01
Population 10–19,999	0.89^b^	0.93	0.97
Population 20–49,999	0.89^a^	0.93	0.95
Population 50,000+	0.92	0.89	1.03
***Accessibility***^e^			
Ttt municipal centre (average, minutes)	1.00	1.00	1.00
Hospital in municipality	0.83^c^	0.79^c^	1.05
Ttt local hospital^f^	1.02^c^	1.02^c^	1.00
Ttt local+ hospital^f^	0.99^b^	0.98^c^	1.01^b^
Ttt regional hospital^f^	1.00	1.00	1.00
***Capacity***			
Nurses in LTC	1.03^a^	1.08^c^	0.96^c^
Relative size home care (%budget/10)	1.03^a^	0.96^c^	1.07^c^
Nursing home beds (per 100 pop 80+ years)	0.98^c^	1.00	0.98^c^
**Hospital level**			
***Capacity***			
Hospital beds	0.99	0.92^c^	1.09^c^
Constant	0.91	5.1^c^	0.20^c^

^a^Significance at 0.1 level, ^b^Significance at 0.05 level, ^c^Significance at 0.01 level

^d^Mean of 100 20% samples. Four levels: Decedent (N = 69,887) within Year (2003–2011) within Municipality (*N* = 423–428) within Hospital district (*N* = 43)

^e^Ttt, Travel time to

^f^Measured in units of 15 min

Our results suggest that the likelihood of home death increased with the capacity of home services, in form of the relative budget size of home care, and decreased with the bed capacity of nursing homes. Furthermore, the likelihood of a nursing home death versus a hospital death was not affected by nursing home bed capacity, but it decreased with the relative size of home care budgets. Higher nursing capacity in municipal LTC services, in the form of full-time positions, increased the likelihood of nursing home death relative to both hospital death and home death. Finally, higher bed capacity within hospitals increased the likelihood of both hospital and home death versus nursing home death, but it had no bearing on the likelihood of home versus hospital death.

In terms of accessibility, living in proximity to a hospital (within a municipality with a hospital or with low average travel time to the local hospital) increased the likelihood of dying in a hospital. Increased average travel time to hospitals with specialized functions increased the likelihood of hospital and home deaths versus nursing home death. No significant associations were found for average travel time to the municipal centre or nearest regional hospital.

Of the context variables included at the municipal level, we found that a higher population proportion of young elderly (67–79 years) increased the likelihood of nursing home death versus hospital death, whereas an increased proportion of elderly in the age group 80–89 years reduced the likelihood of home death versus hospital death. Populations with higher marriage rates were associated with lower likelihood of nursing home death, while higher proportions of female employment increased the likelihood of nursing home death, and also reduced the likelihood of home death versus hospital death. The likelihood of home versus hospital deaths was lower in municipalities with a population between 10,000 and 50,000 compared with the smallest municipalities with a population under 5000.

The results of the individual-level variables gender and age were consistent with findings of the descriptive statistics, i.e. that the likelihood of home death was lower for women than men and decreased with age (except for the elderly population aged 89–90 years having a lower likelihood of home death versus hospital death than those aged 90+ years), while the opposite relationships were found for nursing home deaths. Unmarried people were more likely to die in nursing homes than married, widowed and divorced people. Unmarried people were also more likely to die at home as opposed to hospital than married and widowed persons. The results for CoD showed that the likelihood of nursing home death was very high for dementia and high for cancer and respiratory diseases but low for diseases of the circulatory system and external causes. The relative risk of home death compared with that of hospital death was higher for dementia, circulatory diseases and external causes, and lower for cancer and respiratory diseases.

## Discussion

Similar to many high-income countries, there was a significant decrease in the rate of hospital deaths and an increase in nursing home deaths in Norway in the period under study. This trend can partly be attributed to demographic and epidemiological shifts affecting the composition of decedents. More female deaths, higher age at death and fewer deaths caused by circulatory diseases were some of the underlying causes [8]. This study confirmed the importance of individual factors such as age, gender, marital status and CoD as predictors of PoD, but the main contribution of this study was its exploration of the relevance of contextual and system-level determinants.

### PoD and service system capacity and accessibility

Accessibility and capacity of services were clearly associated with PoD. The results revealed both expected patterns of ‘pull factors’, as well as indicated service interactions affecting PoD. These are summarized in Table 5.

**Table 5:** Pull factors and service interactions

	**Service interactions**
**Pull factors towards home death**	
- Relative size of home care budget	Reduce the relative risk of nursing home versus hospital death
**Pull factors towards hospital death**	
- Nearness to local hospital	
- Distance to hospital with additional functions	Reduce the relative risk of nursing home versus home death
- Hospital beds	Reduce the relative risk of nursing home versus home death
**Pull factors towards nursing home death**	
- Nursing home beds	Reduce the relative risk of home versus hospital death
- Nurses in LTC	Reduce the relative risk of hospital versus home death

*LTC* long-term care

As expected, hospital accessibility (living close to a local hospital) and capacity (somatic bed rate) increased the likelihood of hospital death. High capacity of nursing home beds increased the likelihood of nursing home death, while a high proportion of LTC spending on home care increased the likelihood of home death. More surprising, perhaps, was the finding that long travel time to the nearest hospital with more specialized functions appeared to serve as a pull factor towards hospital death. The most likely explanation for this finding is that patients potentially in need of specialized hospital services are more likely to be admitted as a precautionary measure and at an earlier time if the travel time is high. The capacity of nurses in LTC was found to function as a pull factor towards nursing home death. Because we were not able to separate nurses by their place of work (home care or residential care), it is unclear whether this reflects a higher capacity of nurses in nursing home care or a general high nurse capacity.

The PoD occurs within a closed health service system with a zero-sum outcome, because people end up dying only in one place, and the system factors that increase the likelihood of death in one particular place will be perfectly balanced by a corresponding total reduction in the likelihood of death in the alternative locations. What is interesting, notably from a policy perspective, is how these effects are balanced between the alternative locations. For instance, while increased hospital capacity reduced the likelihood of dying in a nursing home, not only compared with dying in a hospital, but also compared with dying at home, it did not affect the likelihood of death at home versus in hospital. Similarly, it can be observed that higher relative spending on home care reduced the likelihood of dying in a nursing home compared with dying in a hospital. When these effects are observed together, a pattern of service interdependence or interaction emerges, wherein hospital and home care resources together serve as an alternative to EoL care in nursing homes.

The two ‘pull factors’ towards nursing home death influenced the relative risk of home death versus hospital death differently. Bed capacity in nursing homes affected the likelihood of home death, but not that of hospital death, and decreased the relative risk of home death versus hospital death. Moreover, nursing capacity in LTC appeared to influence hospital deaths the most, increasing the relative risk of home death versus hospital death. One possible explanation for these differences may be that higher bed capacity contributed to more elderly people residing permanently in nursing homes and therefore increasing the likelihood of dying in nursing homes as opposed to a home setting, while higher rates of nursing staff in municipalities contributed to nursing homes being real alternatives to hospitals as EoL care settings. In 2011, approximately 40% of all people who died in nursing homes were on short-term stay, including observation, treatment, rehabilitation, respite and day stay. Nearly one-fifth of people dying in hospital the same year were admitted from a nursing home, of whom 53% were admitted after a short nursing home stay.

The current policy in Norway is to integrate and strengthen the competency in EoL care in the ordinary health and long-term services to ensure good and coordinated patients’ pathways for all dying patients [19]. There is a limited number of dedicated palliative beds and inpatient palliative units in hospitals (110 of approximately 13,340 somatic hospital beds in 2017) and in nursing homes (approximately 440 beds of approximately 40,400 LTC beds in 2017); hospices are almost non-existent and only two of the above-mentioned units refer to themselves as hospices [19]. The Coordination reform of 2012, aimed at strengthening the local health and LTC service provision and reducing the demand for hospital services [20], supports a shift from hospital to nursing home deaths [21]. Home deaths are not common in Norway. National health authorities recommend that palliative care at home is facilitated if patients wish to spend more time at home or die at home [22], but the availability of personnel with special competence in palliative care, such as cancer nurses and mobile palliative teams, varies. Satisfactory palliative care in patients’ homes depends on close collaboration and dialogue between the patient, family, home care nurses and general practitioners [23]. A recent Norwegian study estimated that the potential rate of planned home deaths for community dwellers was 24%, of which only a third of deaths occurred at home [24]. The same research group found that nearly 60% of those dying at home had received domiciliary care some time during the 0–90-day period prior to their death. Moreover, they estimated that no more than 50% of all home deaths were associated with potentially planned palliative and domiciliary care [25]. The likelihood of home death increases with home visits by general practitioners in the last weeks before death; however, few people receive such visits [26].

### International comparisons

A positive association between hospital bed rates and hospital deaths was also found in previous studies [16, 27–30], even when contrasted with home death [11, 14, 31, 32] or nursing home death [14, 33] separately. A negative association between hospital bed rates and home death was also reported [34–37]. Similar to our study, other studies did not find that hospital bed capacity influenced the likelihood of hospital death versus home death [33, 38]. However, contrary to our study, some studies did not find that hospital bed capacity influenced the likelihood of hospital death versus nursing home death [38, 39]. LTC bed availability is typically associated with higher rates of nursing home death [14, 15, 33, 39–41] and lower rates of hospital [16, 29, 30] and home [35] deaths. However, other studies did not find any association between LTC bed availability and nursing home [38] or home [34] death. Few studies included variables capturing availability of home care services. An exception is a recent Japanese study that found a positive association between the proportion of home deaths and home care resources at the municipal level [35]. Comparison of studies is hampered by differences in study populations (e.g. general population, palliative subset, patients with cancer, patients with dementia and elderly people), institutional contexts (e.g. only institutional care), contrasts (e.g. hospital versus all, home versus all and home versus hospital), as well as methodology used (e.g. individual-level data versus aggregated proportions, multinomial or binomial logistic regression and clustering/hierarchical or not). Organizational and structural differences between countries are likely to lead to different results on system variables, as is often the case when several countries are included in the same study [11, 16, 30, 33, 34].

We did not find any study with analyses comparable to our analyses of the effects of travel time to hospital. Urban residency can perhaps serve as a proxy for nearness to hospital. Several studies supported more hospital deaths [11, 15, 30–33, 41–45] and fewer home deaths [13, 27, 34, 35, 46] in urban areas than rural areas. Others found no or mixed effects [11, 13, 30, 33, 34, 39, 40, 44, 45, 47–49].

We identified significant context effects on PoD. We found mixed effects of the age composition variables, probably reflecting that high population numbers in different elderly age segments affect the demand for services differently. The reduced likelihood of nursing home death in municipalities with a high proportion of married adults may reflect different EoL care preferences and/or different informal care capacity. Interestingly, we found a strong effect of female work participation on nursing home death. This could reflect that nursing homes represent labour-intensive services with a high rate of female employment, and in this sense, provide another measure of nursing home capacity, and/or that high female work participation reduces preference for home death, including the informal caregivers’ preferences, which have been previously shown to influence PoD [27].

Finally, the association between PoD and individual characteristics of the deceased found in this study are broadly in line with previous research findings. Lower likelihood of hospital death for women than men is in line with numerous studies on broad decedent populations [16, 43, 50, 51]. Likewise, we found a higher likelihood of nursing home death for women than men, which is also a typical pattern observed in previous studies [40, 43, 51, 52]. Moreover, our study demonstrated the same age effects as other studies, i.e. lower likelihood of hospital death and higher likelihood of nursing home death with higher age, at least among older age groups [43, 44, 50, 53]. While Gruneir et al. [40] found that the relative risk of hospital death versus home death decreases with age, we found, as did Reich et al. [52], that young people are more likely to die at home. Marital status also impacts PoD. We found that married persons had a higher likelihood of hospital death and lower likelihood of nursing home death than unmarried persons, which has also been observed previously [40, 51]. However, our study found that the relative risk of home death compared with hospital death is lower for married than unmarried persons. This finding is in contrast with those of many other studies that observed that being married, or not living alone, is associated with higher likelihood of home death [54]. Our finding could reflect that home death is the exception in Norway, and that being married implies that there is another person in the home to assist with care seeking. Contrary to the main finding in the review by Costa et al. [54], we found a higher likelihood of nursing home death and a lower likelihood of home death versus hospital death for patients with cancer. Cohen et al. [16] also reported mixed results for cancer compared with other causes of death in six European countries.

The pattern of PoD for individual-level variables is likely to vary over time, as shown by Dasch et al. [43], reflecting changes in EoL organization and policy. In Norway, there has been a strong shift from hospital death towards nursing home death for patients with cancer [8], in contrast to some other countries, e.g. there was a shift from hospital to home deaths for patients with cancer in England after 2003 [55]. Norway, in general, has a low proportion of home deaths compared with other countries [10], especially for patients with cancer [13].

### Strengths and limitations

A strength of our study is our comprehensive population dataset, which includes both cross-sectional and time variations in PoD and system characteristics. Another strength is that we explicitly modelled the two-level structure of service organizations and included variables characterizing the services at both levels, while at the same time controlling for individual and contextual factors. Typically, many studies ignore the nested structure of data and clustering of observations at higher levels. This can lead to serious problems of overconfidence in the results on especially higher-level variables because ignored clustering will generally cause standard errors of regression coefficients to be underestimated. Hence, a major strength of our study is that we used a multilevel analytical approach, allowing for variation at all levels. Finally, we applied a multinomial model, i.e. allowing for several contrasts, which provided a more comprehensive picture of system effects and service interactions.

There are some important limitations of this study. First, we only observed PoD and not the place of EoL care. Second, we did not know the quality of services received, or whether the person received special palliative care. Third, we were not able to control for place of residence, and in many cases, the nursing home will also be the place of residence. Fourth, the care needs of dying people and the length of their care needs could be a major consideration in where people die. Unexpected sudden serious health issues such as pain can influence the PoD. We were unable to control for such major influences on the PoD. Finally, this study was part of a larger project on EoL service utilization, which involved complex data linkages and time-consuming negotiations with data owners; thus, the last year covered in our data is 2011. Important changes in the health care system have occurred since then; most importantly, the Coordination reform was introduced in 2012. On analysing the national statistics for PoD during the period 2012–2018, we found an increase in out-of-hospital institutional deaths and a decrease in hospital and home deaths. Hence, this is a continuation of the trend found in our data from before the Coordination reform [8].

## Conclusions

There is substantial variation in the PoD between municipalities and hospital districts in Norway, not only reflecting differences in decedents’ characteristics, but also local circumstances, such as nearness to hospital and service capacities. It has been a core value for palliative care to enable people to make choices about their EoL care and PoD. People generally prefer to die at home, and Norway has a low proportion of home deaths compared with other countries. The proportion of home deaths varies considerably between local communities. Increasing the availability of home care services will likely provide people a more active voice in the decisions tied to their PoD, allowing more people to die at home, if that is what they prefer. In addition, these findings may have important policy implications for the EoL care in other countries with demographic and epidemiological trajectories similar to those in Norway. Further studies in other national contexts should be undertaken to support such conclusions.

## Data Availability

The analysis was based on data from the Norwegian Cause of Death Registry. Data from the registry are available for research projects approved by the registry and those that meet the requirements of the Health Research Act and the Personal Data Act.

## Abbreviations

EoL: End of life
CoD: Cause of death
LTC: Long-term care
NCoDR: Norwegian Cause of Death Registry
PoD: Place of death
RRR: Relative risk ratio
SSB: Statistics Norway
